# Fatty Acid Ethyl Esters in Virgin Olive Oils: In-House Validation of a Revised Method

**DOI:** 10.3390/foods9070924

**Published:** 2020-07-14

**Authors:** Rosa Palagano, Enrico Valli, Matilde Tura, Chiara Cevoli, María del Carmen Pérez-Camino, Wenceslao Moreda, Alessandra Bendini, Tullia Gallina Toschi

**Affiliations:** 1 Department of Agricultural and Food Sciences (DISTAL), Alma Mater Studiorum-Università di Bologna, 47521 Cesena, Italy; rosa.palagano@unibo.it (R.P.); enrico.valli4@unibo.it (E.V.); matilde.tura2@unibo.it (M.T.); chiara.cevoli3@unibo.it (C.C.); tullia.gallinatoschi@unibo.it (T.G.T.); 2 Instituto de la Grasa (CSIC), 41013 Sevilla, Spain; mcperezcamino@ig.csic.es (M.d.C.P.-C.); wmoreda@ig.csic.es (W.M.)

**Keywords:** fatty acid ethyl esters, virgin olive oil, off-line HPLC-GC-FID, sustainable, quality control, PTV, SDG 12

## Abstract

The content of fatty acid ethyl esters (FAEEs) is one of the quality parameters to define if an olive oil can be classified as extra virgin as these compounds are considered markers for virgin olive oils obtained from poor-quality olives. In addition, FAEEs can also be indirect markers to detect soft deodorization treatment. In this study, an off-line HPLC-GC-FID method for determination of FAEEs is presented, revising the preparative step and the GC injector required by the official method (EU Reg. 61/2011). After optimization, the method was validated in-house by analyzing several parameters (linearity, limit of detection LOD, limit of quantification LOQ, robustness, recovery, precision, and accuracy) to determine its effectiveness. Linearity was measured in the 2.5–50 mg/L range; furthermore, intra-day and inter-day precision values were lower than 15%, while the LOD and LOQ were lower than 1 and 1.5 mg/kg, respectively, for all compounds considered. The main advantages of this revised protocol are: (i) significant reduction in time and solvents needed for each analytical determination; (ii) application of HPLC as an alternative to traditional LC, carried with manually packed glass columns, thus simplifying the separation step.

## 1. Introduction

Considering world production of vegetable oils, olive oil is in ninth place with about 3 million metric tons produced during the 2018/19 harvest (USDA, 2019) [1]. Virgin olive oil (VOO) is a product with high commercial value, mainly linked to the presence of healthy components and its sensory characteristics, rendering quality control a highly complex and evolving issue [2] (Clodoveo et al., 2014).

At present, European regulations (CEE Reg. 2568/91 and following amendments) [3] define five parameters to determine and verify the quality of a VOO: Free acidity, peroxide index, specific extinctions in UV, sensory evaluation, and content of fatty acid ethyl esters (FAEEs). Even if this latter parameter was officially introduced by the International Olive Council IOC in 2010 (COI/T.20/Doc. No 28) [4] and by the European Union in 2011 (EU Reg. n. 61/2011) [5], the study of these compounds started many years ago, giving rise to a double meaning in their evaluation (as direct markers of quality and as indirect markers of illegal technological treatment, as explained below).

Detected for the first time by Mariani and Fedeli in 1986 [6], alkyl esters of fatty acid (FAAEs, which include fatty acid methyl and ethyl esters) are formed by degradation and fermentation processes in low-quality olives, such as those that are overripe, damaged, or stored in poor conditions before processing (Biedermann et al., 2008) [7]. As a consequence, the degradation of pectins by endogenous pectin methylesterases and the aerobic metabolism of microorganisms can lead to production of short-chain alcohols, namely methanol and ethanol, respectively (Conte et al., 2019) [8]. The esterification of these compounds with free fatty acids, produced by lipolysis of triacylglycerols, leads to the development of methyl and ethyl esters of fatty acids. It is a typical second-order reaction that takes place in an acidic medium and is catalyzed by enzymes (Pérez-Camino et al., 2008) [9]. Moreover, the formation of these molecules can continue during storage of oil, but this phenomenon is strictly linked to the presence of free alcohols and fatty acids formed as a consequence of the fermentation of olives (Conte et al., 2014; Squeo et al., 2019) [10,11].

Pérez-Camino and co-workers (Pérez-Camino et al., 2002) [12] proposed an analytical method to determine the content of FAAEs in vegetable oils, showing the relation between their content and quality of olive oil: FAAEs were more abundant in olive oils obtained from damaged olive fruits. This result, subsequently confirmed by other studies (Mariani & Bellan, 2008; Biedermann et al., 2008; Pérez-Camino et al., 2008) [7,9,13], suggested the possibility to introduce this parameter in the quality control of VOOs, as it represents a direct indicator of the degradation and fermentation of olives, and is thus linked to the presence of fermentative sensory defects (Gomez-Coca et al., 2012; Di Serio et al., 2017) [14,15]. However, as fermentation processes are linked only to the content of FAEEs, in 2013 (EU Reg. 1348/2013) [16], the legal limit for this parameter was changed to exclude quantification of FAMEs (fatty acid methyl esters). At present (EU Reg. 2019/1604) [17], even if the amount of FAEEs in extra VOO (EVOO) is generally lower, the limit was set at 35 mg/kg of oil, in order to use caution.

The second relevance of the content of FAEEs is related to soft deodorization, a technological process applied to VOOs with weak organoleptic defects in order to remove or reduce these off-flavors. The blending of soft deodorized oil with EVOO and possible commercialization of this blend labelled as top-quality grade (EVOO) is an illegal practice. As the technological conditions of temperature and pressure applied in the soft deodorization process are “mild” and avoid the formation of typical markers of refining (such as stigmastadienes or trans isomers of fatty acids), in treated oils, it is very difficult to detect this type of fraud (Valli et al., 2013) [18]. Many studies (Pérez-Camino et al., 2008; Jabeur et al., 2015) [9,19] have reported that the content of FAAEs is not affected by the soft deodorization process, thus allowing for indirect detection of this illegal blend as oils subjected to soft deodorization are usually rich in FAEEs that are not significatively removed or altered by this treatment.

The official method for determination of the content of FAAEs (EU Reg. n. 61/2011) [5] is based on the addition of suitable internal standards to the oil and fractionation by traditional liquid chromatography on a hydrated silica gel column. After recovery of the eluted fraction, this is analyzed by capillary gas chromatography. It should be pointed out that the official method requires a large volume of solvents and entails a very long and complex preparative procedure. For these reasons, the method was revised by the IOC (COI/T.20/Doc. No 31) [20], reducing the amount of silica and eluent mixture. More recently, EU Reg. 2019/1604 [17] introduced the possibility to replace n-hexane with iso-octane. A further revision found in the literature involves the injection technique used for GC analysis: A programmed temperature vaporizer (PTV) injector used as an alternative to the on-column one required by the regulation gives comparable results in analysis of real-world samples (Purcaro et al., 2015) [21]. Moreover, other analytical techniques have been tested as alternatives to the official method. Regarding chromatographic techniques, the first method proposed in 2002 (Pérez-Camino et al., 2002) [12], and later slightly modified by the same research group (Pérez-Camino et al., 2008; Gomez-Coca et al., 2012) [9,14], was based on isolation of the fraction under study from the oil by solid-phase extraction (SPE) and subsequently analyzed by GC-FID. In 2008 (Biedermann et al., 2008) [7], an on-line LC-GC-FID method was optimized for analysis of methyl/ethyl oleate and selected straight chain wax esters. Subsequently, the same approach was used by Küchler and coworkers (Küchler et al., 2014) [22], which reduced the manual sample preparation effort by 90%. More recently, a GC-(EI) MS with a PTV injector coupled to a capillary transfer line with an external thermal extraction unit was optimized and applied to a large set of olive oil samples of different quality (Boggia et al., 2014) [23]. Finally, rapid and non-destructive techniques based on FT-IR (Fourier-transform infrared spectroscopy) (Valli et al., 2013; Squeo et al., 2019; Uncu et al., 2019) [11,18,24], time-domain reflectometry (TDR) (Berardinelli et al., 2013) [25], and near-infrared spectroscopy (NIR) (Garrido-Varo et al., 2017; Cayuela, 2017) [26,27], coupled to chemometric data elaboration, have also been applied with good results. However, as it is well known, in the case of the application of these analytical approaches and specific statistical treatments, large sets of data for calibration and validation steps are needed in order to obtain reliable results.

The aim of the present investigation, developed within the framework of the H2020 OLEUM project, was to evaluate the application of possible improvements to the official method for FAEE determination, which have been recently highlighted (Conte et al., 2019) [8]. In particular, the use of HPLC-UV-Vis as an alternative to traditional liquid chromatography applied in the preparative phase, as well as the use of a PTV injector as an alternative to the required on-column injector OCI, has been investigated. Next, the revised method (off-line HPLC-GC-FID with PTV injector) was validated in-house by evaluating selected parameters (linearity, limit of detection LOD and limit of quantification LOQ, robustness, intra-day and inter-day precision, accuracy, and recovery). The use of an off-line HPLC-GC approach has the potential to represent a good compromise between traditional liquid chromatography foreseen in the official IOC and European Community EC methods and the application of an on-line LC-GC system that would require expensive instrumentation, which is not affordable for most laboratories.

## 2. Materials and Methods

### 2.1. Reagents and Chemicals

Diethyl ether (purity ≥ 99.8%), *n*-hexane (purity ≥ 95%), *n*-heptane (purity ≥ 99%), and tert-butyl methyl ether (MTBE, purity ≥ 99.8%) were supplied by Sigma-Aldrich, Inc. (St. Louis, MO, USA). Sudan I (1-phenylazo-2-naphthol, CAS number 842-07-9), methyl heptadecanoate (analytical standard, CAS number 1731-92-6), ethyl palmitate (analytical standard, CAS number 628-97-7), ethyl oleate (analytical standard, CAS number 111-62-6), ethyl linoleate (analytical standard, CAS number 544-354), ethyl stearate (analytical standard, CAS number 111-61-5), and silica gel (high-purity grade, Davisil Grade 62, particle size 60-200 mesh) were purchased from Sigma-Aldrich, Inc. (St. Louis, MO, USA).

### 2.2. Determination of FAEEs by Off-Line HPLC-GC-FID

Sample preparation: 75 mg of oil was weighed and dissolved in 0.50 mL of internal standard solution (methyl heptadecanoate C17:0, 0.01% in *n*-heptane) and 0.75 mL of Sudan I solution (0.00025% in *n*-hexane). The sample was then filtered with a polyamide filter (0.20 μm) and transferred to a vial for HPLC.

Collection and separation of the FAEE fraction: Extraction and collection of the fraction containing FAEEs were carried out using an HPLC-UV-Vis instrument (Agilent 1260 Infinity II; Santa Clara, CA, USA) equipped with a silica column (Luna^®^, particle size 5 μm; length 25 cm; internal diameter 4.6 mm; Phenomenex, Torrance, CA, USA). The isocratic mode was applied with n-hexane/MTBE (95:5 *v/v*) as a mobile phase. The flow was set at 1 mL/min and the UV detector at 460 nm to monitor the dye (Sudan I) elution. A 100 µL aliquot of the sample was injected and the FAEE fraction was manually collected until the beginning of Sudan elution (by monitoring the elution from the real-time window of the HPLC software). Next, the collected solution was dried under a gentle nitrogen flow to concentrate the FAEEs. Following this, FAEEs were recovered with 300 μL of *n*-heptane, the same solvent established in the official method, for subsequent GC-FID analysis.

GC-FID analysis: This step was carried out using a GC-FID (Trace 1300; Thermo Fisher Scientific, Waltham, MA, USA) equipped with both a PTV and on-column injector and a capillary column TG-5SILMS (length 15 m, internal diameter 0.32 mm, film thickness 0.25 μm, stationary phase 5% diphenyl/95% dimethyl polysiloxane; Thermo Fisher Scientific, Waltham, MA, USA). The oven program temperature was according to EU Reg. 61/2011: 80 °C for 1 min; increasing by 20 °C/min until reaching 140 °C; increasing by 5 °C/min until 335 °C, and maintaining for 20 min. The detector temperature was 350 °C and the injection volume was 1 µL. Helium was used as carrier gas with a flow of 1 mL/min. For PTV injection conditions, the splitless mode with an injection temperature of 70 °C, increasing by 3 °C/s until 300 °C (maintained for 1 min) during the transfer phase, was applied.

Identification and quantification of FAEEs: Peak identification was carried out by directly injecting a solution containing a mixture of standards (ethyl palmitate, ethyl oleate, ethyl linoleate, ethyl stearate) in the GC-FID and comparing the retention times of each compound vs. known standards. Relative retention times were also calculated by measuring the ratio between the retention time of each compound and that of the internal standard. The quantification step was carried out according to EU Reg. 61/2011 [5]. FID response was assumed to be equal for all compounds, and no correction for the response was applied.

### 2.3. In-House Method Validation

The parameters considered for in-house validation of the method were linearity, LOD and LOQ, recovery, robustness, accuracy, and intra-day and inter-day precision. For evaluation of these parameters (except for accuracy and intra-day precision), the sample was prepared following the steps described above (see Section 2.2) but using refined olive oil and replacing the internal standard solution with 0.50 mL of a mixture of standards solution in *n*-hexane. The use of refined olive oil was preferred to avoid the presence of FAEEs that could interfere with the separation. The standards used, chosen according to the usual FAEE composition in VOO, were ethyl palmitate, ethyl stearate, ethyl oleate, ethyl linoleate, and methyl heptadecanoate (as internal standard) dissolved in *n*-hexane at different approximate concentrations: 0.0025 mg/mL (C1), 0.005 mg/mL (C2), 0.010 mg/mL (C3), 0.025 mg/mL (C4), and 0.050 mg/mL (C5).

To evaluate the linearity of the method, all the solutions of the mixture of standards were analyzed, and the relation (expressed as R^2^) between the areas measured for each compound and their concentration was determined. Moreover, the linearity was investigated by using the lack-of-fit test according to Sanagi et al. [28]. This test is based on the assumption that the residual sum of squares is separated into pure error (from repeated points) and lack-of-fit (deviation from linearity) sum of squares. The regression model is acceptable if the *F_reg_* value is higher than the critical value F(α,1,p(n – 1)), while the linear model is acceptable if the *F_lof_* value is lower than or equal to the critical value F(α, p – 2,p(n – 1)), being p and n, the number of calibration points and the replicates, respectively. Details on the formulas used for the lack-of-fit test (sum of squares, degrees of freedom; mean squares and Fisher ratio) are extensively described by Sanagi et al. [28].

The LOD and LOQ for each compound were evaluated considering the standard deviation of the lowest acceptable concentration of the analyte (in this case C1) and the slope of the calibration function of each molecule (González & Herrador, 2007) [29].

For recovery, the solutions of the mixture of standards C3, C4, and C5 were analyzed by applying the revised procedure (off-line HPLC-GC-FID with PTV injector) and direct gas chromatographic on-column injection. The results obtained for ethyl esters and methyl heptadecanoate (used as an internal standard) were then compared and recovery percentages were calculated. Specifically, for each tested concentration (C3, C4, and C5 concentrations), the ratio between the area of the analyte added to the oil sample, processed by HPLC fractionation and analyzed by GC, and the area of the same analyte assessed by direct GC injection was calculated and multiplied by 100. Each processed area (area of the analyte in the samples processed by HPLC) was calculated considering the area of the analyte multiplied by the ratio between the concentration of the analyte by direct injection and the concentration of the analyte processed. One-way ANOVA, test LSD-Fisher, *p* ≤ 0.05 was applied to verify if significant differences were found among the different tested concentrations.

For robustness, the standard solution C4 was analyzed with the revised protocol, and the mobile phase flow for the HPLC analysis was changed from 1 to 0.7 mL/min. The data obtained for each standard were elaborated (Student’s *t*-test, *p* ≤ 0.05). For inter-day precision, one sample (solution C4) was analyzed by applying the proposed method on three different days. Next, the area values of each compound measured on three different days were evaluated (one-way ANOVA, *p* ≤ 0.05).

To study the accuracy and intra-day precision of the revised method, three VOOs with a different FAEE content (sample LC–low content: EVOO with a content of FAEEs under the legal limit; sample MC–medium content: Non-EVOO with a medium content of FAEEs beyond the legal limit; sample HC–high content: Non-EVOO with a content of FAEEs much higher than the legal limit) were analyzed by both the official method (according to EU Reg. 61/2011) [5] and the revised one. The results with the two methods were compared (Student’s *t*-test, *p* ≤ 0.05) to verify the effectiveness of the proposed protocol. The intra-day precision was expressed as RSD. [Dataset] is available according to Palagano et al., 2020 [30].

## 3. Results and Discussion

### 3.1. Development of the Method

The starting point of the present research was the need to find an analytical solution for determination of FAEEs in VOO that can overcome some of the drawbacks of the official method and, at the same time, would be applicable by the majority of laboratories and industries. In fact, on-line approaches require expensive instrumentation and specific analytical skills that are not always available, especially outside an academic context.

In this study, an off-line combination of HPLC-GC-FID as an alternative to the official method for determination of FAEEs in VOOs was developed. The preparative phase, i.e., extraction and separation of the fraction containing FAEEs, was carried out using HPLC-UV-Vis in place of traditional liquid chromatography applied in the official method. Volumes of the solvents and time requested for each determination applying the off-line combination of HPLC-GC-FID were 40 mL and 2.5 h in place of 350 mL and 6 h needed for the official method, respectively. The specific analytical conditions adopted were chosen in order to apply the same analytical principle of the official method: Normal-phase liquid chromatography in isocratic mode. For this reason and following previous reports (Biedermann et al., 2008; Küchler et al., 2014) [7,22], a silica HPLC column and a slightly polar mixture of solvents (*n*-hexane/MTBE 95:5 *v/v*) as the mobile phase were chosen. The analytical conditions applied herein lead to elution of both FAMEs and FAEEs, even though a legal limit is defined only for the latter. For this reason, only the results on FAEEs will be presented and discussed herein.

As in the official method, in this case, Sudan I was used as a dye to visualize elution of the fraction containing FAAEs as it has an elution time between those of ethyl esters and triglycerides. Therefore, elution of Sudan I indicates the end of elution of the analytes of interest, avoiding the undesired elution of triglycerides (Pérez-Camino et al., 2002) [12]. Consequently, to detect dye elution, a UV-Vis detector was selected and used at a wavelength of 460 nm, which is in the absorption range of this compound. Some attempts to find an alternative to this compound have been previous carried out as it is known to be both toxic and carcinogenic, and its addition to food as a colorant is not permitted (Genualdi et al., 2016) [31]. In particular, α-tocopherol and bixin were tested, but satisfactory results were not obtained, due to different retention times and low solubility in the solvent used for the analysis, respectively. Under standardized elution conditions, to be carefully defined by each user, it might also be possible, even if highly challenging, to avoid the use of Sudan I by defining a set time that is able in which the analytes are eluted and, at the same time, guarantee the absence of triacylglycerols in the fraction collected. Even if the elution of Sudan I, followed by elution of the fraction containing FAEEs, ends after about 8–9 min of analysis (depending on the pressure reached), the total duration of each HPLC analysis was set at 30 min to allow complete elution of triacylglycerols (TAGs) prior to the next injection, avoiding their permanence in the column. Following this, the fraction containing FAEEs was analyzed by GC-FID with the PTV injector. This represents the only difference in the gas chromatographic step between the proposed method and the official one for which an OCI is needed. The use of an OCI is required for many applications in the analysis of fats and oils to avoid discrimination effects and reduce degradation of labile compounds (Purcaro et al., 2015) [21], but it is not widely applied in quality control laboratories, and, thus, an alternative is needed. The PTV injector represents a more versatile option as the temperature control is time-programmed and the injection mode (split/splitless) can be optimized according to the specific application (Engewald et al., 1999) [32]. Moreover, PTV injectors can be adapted, with some technical expedients, to mime an OCI injection. It should also be highlighted that a thermodesorption system in connection with PTV has been used for the separation of FAEEs (Boggia et al., 2014) [23]. However, to avoid the use of a system that is not widely diffuse, an HPLC separation was developed as it is much more widely available.

Identification of compounds was carried out by injecting the analytical standard of each molecule, and the relative retention time (RRT) was calculated (Table 1) considering the mixture of standards (C1–C5). Applying the revised method, the total time required for each analytical determination was reduced from about 6 to roughly 2.5 h. Considering that the duration of the chromatographic run was the same for the official and revised methods, the reduced time was mainly due to the speeding up of the preparative phase by HPLC that required drying almost 250 mL in a rotary evaporator. Another advantage of HPLC is that manual packing of the glass column for the liquid chromatography is not required (nor is previous heating of silica), thus simplifying this step.

### 3.2. Method Performance Determined with the In-House Validation

After establishing the analytical conditions, the proposed method was validated in-house using specific parameters: Linearity, LOD and LOQ, recovery, robustness, accuracy, and inter-day and intra-day precision.

**Figure 1:**
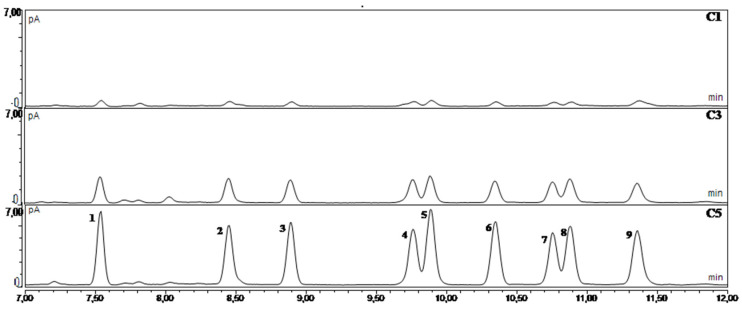
Overlay of chromatograms obtained from the analysis of the solutions of standards C1, C3, and C5 applying the revised method. Compounds identification: (1) Methyl palmitate, (2) ethyl palmitate, (3) methyl heptadecanoate (internal standard), (4) methyl linoleate, (5) methyl oleate, (6) methyl stearate, (7) ethyl linoleate, (8) ethyl oleate, (9) ethyl stearate.

For linearity, the mixture of standards was analyzed at five different concentrations: Peak area vs. analytes concentration showed good correlation coefficients (expressed as R^2^), and the linearity was verified by the lack-of-fit statistical test for all compounds (Table 2). The method showed a linear response between 2.5 and 50 mg/L (Figure 1), which is within a reasonable range in view of the legal limit established for EVOO (EU Reg. 2019/1604) [17].

Results of the lack-of-fit test, in the form of an analysis of variance (ANOVA), are reported in Table 2 for all the considered analytes. The *F_cal_* for the regression are much higher than the corresponding *F_crit_* value, suggesting that all the regression models are acceptable. On the contrary, the *F*_cal_ for the lack-of-fit is lower or equal than the corresponding *F_crit_* value, proving that the linearity is accepted for all the models.

LOD and LOQ values for each FAEE examined were calculated by multiplying the standard deviation of the areas of each compound at the lowest concentration level (in this case, C1), 3 and 10 times, respectively, and then by dividing the result by the slope of the specific calibration curve. All the values obtained for the LOD were lower than 1 mg/kg and those measured for the LOQ were lower than 1.5 mg/kg, in accordance with what has been previously reported (Biedermann et al., 2008) [7].

Recovery percentages, calculated analyzing the mix of standards at three concentrations (C3, C4, C5), for the fatty acid ethyl esters considered and methyl heptadecanoate (used as internal standard) are shown in Table 3, and satisfactory results were obtained. In particular, in the case of the concentration (C4) closer to the legal limit, the recoveries were higher than 94% (Table 3).

Regarding the robustness of the method, the data obtained for all the compounds in the case of a sample injected in HPLC at two different mobile phase flows (1 and 0.7 mL/min) were elaborated, and no significant differences between the two methods (Student’s test, *p* ≤ 0.05) were seen, even though a reduction in the flow rate led to a decrease in pressure and an increase in the elution time of the dye.

To evaluate inter-day precision, comparing the ratio between the area of each compound and the area of the internal standard measured on three different days, no significant differences (one-way ANOVA, *p* ≤ 0.05) were found (Table 4).

For evaluation of accuracy and intra-day precision, and to test the revised method on real-world oil samples, three VOOs with a different FAEE content (low, medium, and high) were analyzed by both the official method and the revised one (off-line HPLC-GC-FID with PTV injector).

In Table 5, the total FAEE content is presented as this is considered by the official method. Moreover, it is also possible to observe the content of single analytes. The total content of FAEEs did not show significant differences (Student’s *t*-test, *p* ≤ 0.05) with the revised method compared to the official one for any sample. Considering these results, the three samples were classified in the same commercial category by both methods: Sample LC was an EVOO, while samples MC and HC were not.

Finally, intra-day precision was expressed as RSD and all the values obtained were lower than 15%, which is considered acceptable for the validation of a new method (Peters et al., 2007) [33].

## 4. Conclusions

A revised protocol, based on an off-line HPLC-GC-FID approach, for FAEE determination in VOOs is presented. The method is based on the application of HPLC-UV-Vis as an alternative to traditional liquid chromatography applied in the preparative phase for FAEE determination and the use of a PTV injector in place of the OCI one required by the official method.

After optimization of working conditions, the method was validated in-house. The data obtained showed good performance in terms of linearity, LOD, LOQ, robustness, intra-day precision, and recovery. No significant differences in terms of total FAEE content were found when the proposed method was tested on VOOs with different amounts of FAEEs, and the results were comparable to those obtained using the official protocol.

The main advantages of this revised protocol are: (i) A significant reduction in time (more than 50%) and (ii) solvents (more than 80%) required for each analytical determination, representing, in accordance with SDG 12 target 12.4, the aim “to reduce the release of chemicals to air, water and soil,” using a more environmentally sustainable and a rapid alternative to the official method; (iii) the application of HPLC as an alternative to traditional liquid chromatography carried out in manually packed glass columns, allowing for the simplification of the technique.

Moreover, the adoption of an off-line HPLC-GC approach could represent a good compromise between what is required by the EC and IOC official methods (traditional liquid chromatography) and the application of an on-line LC-GC system that requires expensive instrumentation, which is not affordable for most laboratories.

These characteristics make the method proposed herein exploitable by control laboratories and industry, satisfying the needs of cost reduction and work optimization. In order to confirm and strengthen the reliability and good performances of the approach presented herein, and in view of its proposal to normative bodies for possible adoption, inter-lab validation of this method involving several laboratories (also from private industries) is being carried out within the OLEUM project.

## Figures and Tables

**Table 1:** Relative retention time (RRT) measured for the fatty acid ethyl esters considered in relation to methyl heptadecanoate (used as internal standard).

Compound	Relative Retention Time (RRT)
Ethyl palmitate	0.95
Ethyl linoleate	1.21
Ethyl oleate	1.22
Ethyl stearate	1.28
Methyl heptadecanoate	1.00

**Table 2:** Results of ANOVA statistics on significance test for regression model and linearity (lack-of-fit test) for all the four considered analytes. Linearity was examined considering the mixture of standards at 5 different concentrations (C1: 0.0025 mg/mL, C2: 0.005 mg/mL, C3: 0.010 mg/mL, C4: 0.025 mg/mL, C5: 0.050 mg/mL). The R^2^ values and the equations for calibration curves of the four compounds were: Ethyl palmitate (EP), R^2^ 0.9765 y = 0.0063x + 0.0273; ethyl linoleate (EL), R^2^ 0.9683 y = 0.0058x + 0.0262; ethyl oleate (EO), R^2^ 0.9588 y = 0.0068x + 0.0255; ethyl stearate (ES), R^2^ 0.9867 y = 0.0066x + 0.0346.

	Source	SS	d.f.	MS	*F_cal_*	*F_crit_*	Conclusion
EP	Regression	0.047754	1	0.047754	463.6	16.3	Regression model accepted
	Lack-of-it	0.002470	3	0.000823	7.9	12.1	Linearity accepted
	Pure error	0.002987	5	0.000103			
EL	Regression	0.038275	1	0.038275	329.1	16.3	Regression model accepted
	Lack-of-it	0.002824	3	0.000941	8.1	12.1	Linearity accepted
	Pure error	0.000582	5	0.000116			
EO	Regression	0.048209	1	0.048209	319.3	16.3	Regression model accepted
	Lack-of-it	0.005476	3	0.001825	12.1	12.1	Linearity accepted
	Pure error	0.000756	5	0.000151			
ES	Regression	0.050871	1	0.050871	860.7	16.3	Regression model accepted
	Lack-of-it	0.001506	3	0.000502	8.5	12.1	Linearity accepted
	Pure error	0.000295	5	0.000059			

Note: SS = sum of squares; d.f. = degrees of freedom; MS = mean squares; *F_cal_* = Fisher ratio; *F_crit_* = critical value of *F*-distribution at α = 0.01.

**Table 3:** Recovery percentages, calculated analyzing the mix of standards at 3 concentrations (C3, C4, C5) for the fatty acid ethyl esters (FAEEs)

Compound	Recovery C3 (%)	Recovery C4 (%)	Recovery C5 (%)
Ethyl palmitate	73.2 ± 3.6 ^c^	101.8 ± 1.3 ^a^	90.4 ± 2.0 ^b^
Ethyl linoleate	72.3 ± 6.9 ^b^	94.9 ± 3.2 ^a^	84.0 ± 0.6 ^a,b^
Ethyl oleate	71.9 ± 1.3 ^c^	94.6 ± 1.9 ^a^	86.6 ± 1.0 ^b^
Ethyl stearate	84.9 ± 2.9 ^c^	102.9 ± 2.9 ^a^	94.1 ± 1.0 ^b^
Methyl heptadecanoate	74.7 ± 3.0 ^c^	98.7 ± 1.6 ^a^	87.7 ± 1.2 ^b^

Considered and methyl heptadecanoate (used as internal standard). All the analyses were carried out in duplicate. Different letters in rows indicate statistically significant differences (one-way ANOVA, test LSD-Fisher, *p* ≤ 0.05).

**Table 4:** Inter-day precision, obtained by adding a mix with the FAEE standards and the internal standard at 0.025 mg/mL (C4) to a refined olive oil, and expressed as the ratio between the area of each compound and the area of the internal standard; analyses were carried out in triplicate on three different days; p-values are calculated according to one-way ANOVA (*p* ≤ 0.05).

Compound	Day 1	Day 2	Day 3	*p*-Value
Ethyl palmitate	1.17 ± 0.06	1.17 ± 0.03	1.11 ± 0.03	0.17
Ethyl linoleate	0.97 ± 0.02	0.95 ± 0.03	0.97 ± 0.02	0.56
Ethyl oleate	1.03 ± 0.02	1.05 ± 0.03	1.06 ± 0.02	0.36
Ethyl stearate	0.91 ±0.12	1.03 ± 0.07	0.99 ± 0.11	0.43

**Table 5:** Mean value (three replicates as regards to the results obtained by the official method; three replicates for sample LC and two replicates for sample MC and HC as regards to the results obtained by applying the revised method) and standard deviation of the content of ethyl oleate and fatty acid ethyl esters measured by both the official method (EU Reg. 61/2011) and revised one (off-line HPLC-GC-FID) on three olive oil samples. Data are expressed as mg/kg of oil. Sample LC–low content: Extra virgin olive oil with a content of FAEEs under the legal limit; sample MC–medium content: Non-extra virgin olive oil with a medium content of FAEEs beyond the legal limit; sample HC–high content: Non-extra virgin olive oil with a content of FAEEs much higher than the legal limit.

Sample	LC		MC		HC	
Compound	Official Method	Revised Method	Official Method	Revised Method	Official Method	Revised Method
Ethyl palmitate	4.1 ± 0.6	7.9 ± 1.4	8.2 ± 0.0	10.3 ± 0.8	11.4 ± 0.5	8.4 ± 0.0
Ethyl linoleate	0.9 ± 0.1	<LOQ	2.0 ± 0.1	<LOQ	5.2 ± 0.1	5.2 ± 1.3
Ethyl oleate	12.0 ± 0.3	9.9 ± 0.5	29.6 ± 0.7	28.4 ± 1.2	63.4 ± 2.1	60.8 ± 4.0
Ethyl stearate	1.2 ± 0.1	<LOQ	<LOQ	<LOQ	2.2 ± 0.3	10.7 ± 3.4
Total FAEEs	18.4 ± 1.1	17.8 ± 1.0	39.8 ± 0.7	38.7 ± 0.4	82.1 ± 2.8	85.1 ± 0.5
